# Neutrophil extracellular traps and oxidative stress in systemic lupus erythematosus patients with and without renal involvement

**DOI:** 10.1186/s13075-024-03454-y

**Published:** 2024-12-19

**Authors:** Lu Liu, Karina de Leeuw, Harry van Goor, Berber Doornbos-van der Meer, Suzanne Arends, Johanna Westra

**Affiliations:** 1 Department of Rheumatology and Clinical Immunology, University Medical Centre Groningen, University of Groningen, Hanzeplein 1, Groningen, 9700 RB The Netherlands; 2 Department of Pathology and Medical Biology, University Medical Centre Groningen, Groningen, The Netherlands

**Keywords:** Neutrophil extracellular traps, Systemic lupus erythematosus, Lupus nephritis, Free thiols, Oxidative stress

## Abstract

**Objectives:**

To investigate the levels of plasma neutrophil extracellular traps (NETs) and free thiols, the latter reflecting systemic oxidative stress (OS), and to explore the relationship between NETs and OS in quiescent systemic lupus erythematosus (SLE) patients with and without renal involvement.

**Methods:**

Plasma levels of NETs and free thiols were measured cross-sectionally in 100 SLE patients with low disease activity (SLEDAI < 5), of whom 73 patients had no renal involvement (non-LN) and 27 patients had lupus nephritis (LN). Additionally, 22 healthy controls (HCs) were included. NETs were measured using a myeloperoxidase-DNA complex ELISA and free thiols were measured using a thiol assay kit.

**Results:**

NETs levels were significantly higher in both non-LN and LN patients compared to HCs (*p* < 0.001, *p* = 0.013), with no difference between the two patient groups (*p* = 0.799). Free thiol levels were not significantly different between groups. Interestingly, NETs were negatively correlated with free thiols in all 100 SLE patients (rho = -0.32) and non-LN patients (rho = -0.38), but not in LN patients. Levels of free thiols were significantly lower in subgroups of patients with estimated glomerular filtration rate (eGFR) < 60, serum creatinine (sCr) ≥ 90, C reactive protein (CRP) levels ≥ 5 and body mass index (BMI) ≥ 30. In multivariable regression, disease duration, NETs levels, and eGFR were independently associated with free thiol levels.

**Conclusions:**

Levels of NETs were increased in quiescent SLE patients. Although free thiol levels did not differ among the groups. The levels of NETs and free thiols were independently associated in SLE patients, suggesting a potential role of OS in NETs formation. Therefore, reducing OS might be an additional therapeutic target for SLE treatment.

**Supplementary Information:**

The online version contains supplementary material available at 10.1186/s13075-024-03454-y.

## Introduction

Systemic lupus erythematosus (SLE) is a heterogeneous autoimmune disease with various clinical manifestations. SLE is more common in African Americans, Hispanics, and Asians than in whites with higher morbidity rates in females of childbearing age [1]. Lupus nephritis (LN) is the most severe organ manifestation of SLE. The pathogenesis of LN is characterized by immune complexes deposition in the kidney, which leads to inflammation. Neutrophil extracellular traps (NETs) are regarded as a source of endogenous nucleic acids leading to autoantibody formation [2, 3]. Also, increased NETs formation has been identified as one of the players in the pathogenesis of LN [4, 5].

NETs are formed by neutrophils which are the most abundant innate immune cells playing an important role in the first line of immune defense against intruding pathogens. Neutrophils migrate to the site of inflammation where they act as phagocytic cells. They can produce cytokines, reactive oxygen species (ROS) and reactive nitrogen species (RNS), which all play a role in neutrophil degranulation and NETs formation [6–8]. NETs formation can occur through two pathways: lytic NETosis, involving cell death with cellular and chromatin disintegration, or via a nonlytic process where nuclear chromatin is expelled from the cell accompanied by granule proteins to trap and kill microorganisms [9]. In SLE, NET formation is enhanced, which may already occur in the early stages of SLE and is correlated with disease severity [10, 11]. In addition, NETs components, such as histones, DNA and granular proteins, may serve as autoantigens in the production of pathogenic immune complexes [12, 13]. Furthermore, NETs induce podocyte detachment, proteinuria and eventually podocyte cell death, while release of NETs-associated cytokines may cause tubuloepithelial cell apoptosis [13]. Besides, increased circulating NET remnants seem to be associated with proteinuria and renal lesions in patients with LN [14].

ROS/ RNS are generated by incomplete oxygen reduction during cellular metabolism processes [15]. Oxidative stress (OS), resulting from disequilibrium between the synthesis and neutralization of ROS/ RNS, is considered to contribute to the pathogenesis of SLE [16]. The interaction of OS with carbohydrates, lipids, proteins and nucleic acids in SLE initiates autoimmunity and tissue damage through disturbances in immunometabolism, aberrant production of NETs and affecting cell death signals, including nonphysiological (necrotic) or regulated (apoptotic) pathways [16–18]. Free thiols are organosulfur compounds that carry a sulfhydryl (R-SH) moiety, which reliably reflect systemic OS by participating in the reduction of oxidative modification and being readily oxidized by reactive species [19–21]. Of note, diminished free thiol levels indicate higher levels of ROS and therefore an unfavorable redox state [22]. A long-term follow-up study in patients with LN revealed that free thiols are stable compounds that are associated with disease activity [19]. It is known that ROS/ RNS play an important role in NETs release and treatment of neutrophils with resveratrol, an antioxidant, showed inhibition of NETs release after stimulation with different inducers of NETs formation [23]. Treatment of lupus-prone MRL-lpr mice with the mitochondria-targeted antioxidant MitoQ, led to reduced neutrophil ROS/ RNS and NETs formation [24].

Therefore, to further investigate the interaction between NETs and OS we assessed levels of NETs and OS (as reflected by plasma free thiols) in quiescent SLE patients with and without history of LN compared to healthy controls (HCs). Furthermore, the relation of free thiols and NETs with clinical and biological biomarkers was assessed.

## Material and methods

### Study population

The present cross-sectional analysis was performed in SLE patients from a prospective cohort study conducted at the University Medical Centre Groningen. For this study, 100 SLE patients, of whom 73 patients had no renal involvement (non-LN) and 27 patients had a history of LN, and 22 HCs were included. SLE patients were eligible if they met the SLICC criteria [25]. LN was classified based on the International Society of Nephrology/Renal Pathology Society (ISN/RPS) classification [26]. Disease related information and participants’ demographics were retrieved from medical records. Disease activity was assessed with the systemic lupus erythematosus disease activity index (SLEDAI) [27]. In our cohort, all enrolled patients were in remission, as defined by SLEDAI < 5. The study was approved by the local ethics committee (METc: 2015.313 & 2014.081) and conducted in line with the Declaration of Helsinki. All patients provided written informed consent prior to participation.

### ELISA for MPO-DNA complexes

Free circulating plasma NETs were measured as MPO-DNA complexes in plasma via ELISA. Plasma was isolated from whole blood samples by centrifugation and stored at -20°C until further processing. We adapted the recently published protocol by Matta et al. [28]. Plates were coated in coating buffer containing 2.5µg/ml of anti-MPO antibody (Abcam, Ab 25989). Plates were subsequently blocked with 5% BSA and 5% Normal Rat Serum (Thermo Fisher Scientific), after which diluted plasma was added in duplicate for every participant. After incubation, anti-DNA POD detection antibody (Roche) was added. Color reaction was developed with 3,3’, 5,5; -tetramethylbenzidine (TMB, Sigma-Aldrich). Plates were scanned at 450–575 nm in a spectrophotometer. As an internal standard, a serial dilution of NET-containing supernatant of phorbol-12-myristate-13-acetate (PMA)-stimulated neutrophils from a healthy donor was used. Samples with values below the lower limit of detection (< 0.15) were coded as 0.1.

### Free thiols

Plasma levels of free thiols (μmol/L) were measured using the commercial Measure-iT Thiol Assay Kit (Thermo Fisher Scientific #M30550), following the manufacturers’ instructions with minor modifications.

### Statistical analysis

Data are presented as mean ± standard deviation (SD) for normally distributed and median with interquartile range (IQR: p25-p75) for non-normally distributed continuous variables and number of patients (percentage) for categorical variables. Demographic, clinical, laboratory characteristics and medication use were compared between SLE patients with and without renal involvement using Independent Samples t-test, Mann–Whitney-U test and Chi-Square test as appropriate. Overall differences in biomarkers between the three groups (non-LN, LN and HC) were analyzed using Kruskal–Wallis test and, in case of *P*-value < 0.05, followed by Mann–Whitney U test for pairwise comparisons. Biomarkers were compared between subgroups of SLE patients stratified according to clinical characteristics using Mann–Whitney U test. Spearman correlation coefficients were used to analyse the association between biomarkers in patients with SLE. Of demographic, clinical and laboratory parameters univariable logistic regression was performed with free thiol levels and Log10 (NETs + 1) as dependent variables. The explained variance of clinical and laboratory characteristics was explored using the Nagelkerke R^2^. To evaluate which variables were independently associated with free thiols, variables with *P*-values of < 0.05 in univariable logistic regression were selected for a multivariable logistic regression model (forward selection). Afterwards, the enter method was performed to confirm the significant variables from the forward method. Inclusion of variables for multivariable modelling was checked for multicollinearity and was also based on clinical relevance.

SPSS (version 28.0.1.0) and GraphPad Prism 9 were used for data analysis and graphics. *P*-values < 0.05 were considered statistically significant.

## Results

### Patient characteristics

Characteristics from 100 SLE patients, of whom 27 patients had a history of LN, are depicted in Table 1. LN classification is shown in supplementary Table 1, half of the patients (52%) had class IV. Overall, most SLE patients were female (84%), mean age was 49 ± 11 years, median disease duration was 11 (IQR 6–22) years and disease activity was low (median SLEDAI of 1.4, IQR 0–2). Levels of serum creatinine (sCr), Triglyceride (TG), and proportion of proteinuria positive patients were significantly higher in the LN group compared to non-LN, while levels of haemoglobin (Hb), lymphocytes total amount, and estimated glomerular filtration rate (eGFR) were significantly lower in the LN group compared to non-LN (Table 1).

**Table 1:** Demographic, clinical, laboratory characteristics and medication use of the total group of SLE patients and stratified for renal involvement

	SLE (*n* = 100)	non-LN (*n* = 73)	LN (*n* = 27)	*P-value*^***a***^
Gender, female	84 (84%)	62 (85%)	22 (81%)	0.760
Age, years	49 ± 11	50 ± 14	47 ± 14	0.443
Disease duration, years	11 (6–22)*	11 (6–21)*	12 (5–22)	0.829
BMI, kg/m^2^	25 (23–30)*	25 (23–29)*	26 (24–32)	0.419
Systolic blood pressure, mmHg	120 (110–137)	120 (110–139)	120 (110–130)	0.916
Diastolic blood pressure, mmHg	75 (70–80)	75 (70–80)	78 (70–80)	0.646
Alcohol	20 (20%)**	14 (19%)**	6 (22%)*	0.999
Smoking	25 (25%)**	20 (27%)**	5 (19%)*	0.306
Hypertension	21 (21%)*	15 (21%)	6 (22%)*	0.785
Diabetes	7 (7%)**	5 (7%)*	2 (7%)*	0.673
Obesity	27 (27%)*	16 (22%)*	11 (41%)	0.082
*Laboratorial measurements*				
Thrombocytes, 10^9/L	254 ± 72*	253 ± 78*	256 ± 54	0.972
Hemoglobin, mmol/L	8.1 ± 0.9	8.2 ± 0.9	7.8 ± 0.8	*0.037*
Lymphocytes total amount, 10^9/L	1.4 ± 0.6	1.4 ± 0.6	1.1 ± 0.4	*0.016*
Neutrophils total amount, 10^9/L	4.1 (3.0–5.8)	4.1 (3.0–5.8)	3.9 (3.1–5.9)	0.795
Leukocytes, 10^9/L	6.3 (4.7–8.0)	6.3 (4.9–8.1)	5.8 (4.6–7.4)	0.357
C-reactive protein, mg/L	1.7 (0.7–5.0)*	2.0 (0.8–6.0)*	1.0 (0.4–3.3)	0.071
eGFR, ml/min	87 ± 23	90 ± 21	79 ± 27	*0.043*
Serum creatinine, µmol/L	73 (63–83)	70 (62–80)	78 (72–95)	*0.011*
Albumin, g/L	43 (41–45)**	43 (41–45)**	43 (40–44)*	0.288
Proteinuria positive	11 (11%)	3 (4%)	8 (30%)	*0.001*
Proteinuria, if positive, g/24 h	0.59 (0.40–0.64)	0.40 (0.40–0.52)	0.60 (0.43–0.77)	0.442
Cholesterol, mmol/L	4.3 (3.8–5.0)*	4.3 (3.7–4.8)*	4.6 (4.0–5.1)	0.188
TG, mmol/L	1.4 (1.1–1.9)¶	1.2 (0.8–1.5)¶	1.7 (1.3–2.5)**	*0.006*
LDL-C, mmol/L	2.5 (2.1–3.1)*	2.4 (2.0–3.0)*	2.6 (2.3–3.3)	0.143
HDL-C, mmol/L	1.5 (1.2–1.8)*	1.5 (1.3–1.8)*	1.4 (1.1–1.9)	0.323
ALAT, U/L	19 (14–27)	19 (14–28)	19 (15–26)	0.744
anti-dsDNA titer, IU/mL	4 (1–13)	4 (1–11)	5 (1–19)	0.264
Complement 3, g/L	1.03 (0.90–1.18)	1.02 (0.90–1.17)	1.07 (0.93–1.23)	0.834
Complement 4, g/L	0.18 (0.13–0.23)	0.17 (0.13–0.23)	0.19 (0.14–0.24)	0.630
*Medication use*				
Hydroxychloroquine	80 (80%)	59 (81%)	21 (78%)	0.467
Prednisolone	37 (37%)	21 (29%)	16 (59%)	*0.005*
Aspirin	12 (12%)	9 (12%)	3 (11%)	0.587
NSAID	24 (24%)*	23 (32%)*	1 (4%)	*0.002*
ACE-I or ARB	33 (33%)	13 (18%)	20 (74%)	< *0.001*
Azathioprine	14 (14%)	5 (7%)	9 (33%)	*0.002*
Mycophenolate mofetil	14 (14%)	9 (12%)	5 (19%)	0.311
Methotrexate	8 (8%)	8 (11%)	0	0.072
Leflunomide	1 (1%)	1 (1%)	0	0.730
Cyclophosphamide	2 (2%)	1 (1%)	1 (4%)	0.469

*Abbreviations*: *non-LN* SLE without renal involvement, *LN* Lupus nephritis, *BMI* Body mass index, *TG* Triglyceride, *eGFR* Estimated glomerular filtration rate, *LDL-C* Low-density lipoprotein-cholesterol, *HDL-C* High-density lipoprotein-cholesterol, *ALAT* Alanine aminotransferase, *NSAID* Non-steroidal anti-inflammatory drugs, *ACE-I* Angiotensin-converting enzyme inhibitors, *ARB* Angiotensin receptor blockers, *anti-dsDNA* Anti-double stranded DNA

^a^represents comparison of patients with non-LN and LN

Parameters are presented as mean ± SD, median (IQR) or number (%) of patients; Missing data * ≤ 7%, **7–17%, ¶ > 17%; Positive proteinuria is defined as > 0.3 g/24 h

Concerning treatment, non-LN patients used significantly more often prednisolone and non-steroidal anti-inflammatory drugs (NSAID), while LN patients used significantly more often angiotensin-converting enzyme inhibitors or angiotensin receptor blockers and azathioprine (Table 1).

### Levels of NETs and free thiols in groups

**Fig. 1:**
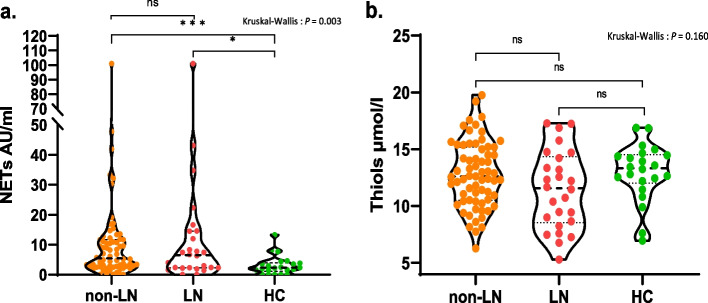
Comparison of NETs and free thiol levels in SLE patients with and without renal involvement and healthy controls (HCs). Legends 1. (**a**) Plasma NETs levels are shown per group (**b**) Plasma free thiol levels are shown per group. Line in each violin plot represents median with interquartile range; *P = 0.013; ***P < 0.001; ns, not significant; non-LN, SLE without renal involvement; LN, lupus nephritis; NETs, neutrophil extracellular traps

Overall, plasma levels of NETs were significantly different between the three groups (*p* = 0.003). Pairwise comparisons showed that the levels of NETs were higher in non-LN (median 5.5, IQR 2.8–11.8) and LN (median 6.5, IQR 2.3–12.0) compared to HCs (median 2.3, IQR 1.5–3.8) (*p* < 0.001, *p* = 0.013 respectively), while no significant difference was observed between non-LN and LN (*p* = 0.799). Levels of free thiols were not significantly different between the groups (*p* = 0.160); non-LN (median 12.6, IQR 10.6–15.0) LN (median 11.6, IQR 9.0–13.4), HCs (median 13.3, IQR 12.3–14.4), although HCs tended to have some higher levels of free thiols, reflecting less OS (Fig. 1).

**Fig. 2:**
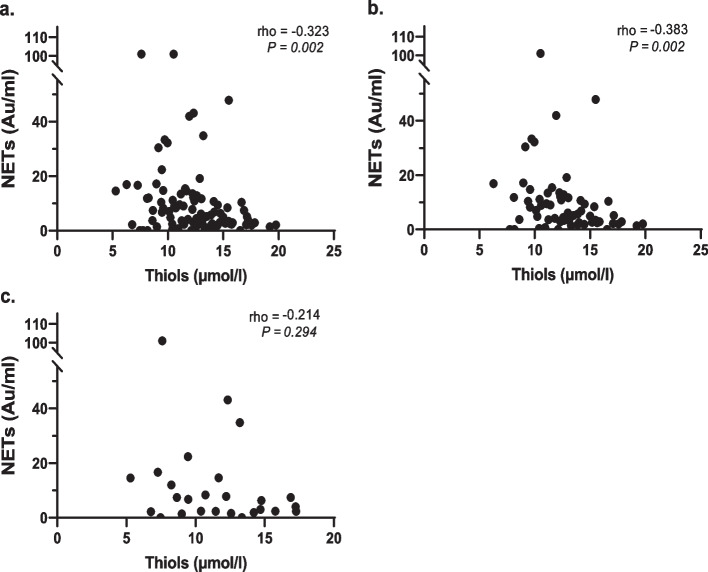
Correlations between levels of NETs and free thiols in the total group of SLE patients and stratified for renal involvement. Legends 2. Correlations between levels of NETs and free thiols. (**a**) All SLE patients; (**b**) SLE patients without renal involvement; (**c**) LN patients; rho, Spearman correlation coefficient; ns, not significant; NETs, neutrophil extracellular traps; LN, lupus nephritis

In all SLE patients, levels of NETs were significantly inversely correlated with levels of free thiols (rho = -0.32), indicating that patients with higher levels of NETs had more OS. After stratifying the patients, the same correlation was found in non-LN group (rho = -0.38), but not in the patients with a history of LN (Fig. 2). NETs levels were low and not negatively correlated to thiols in HCs (data not shown).

**Fig. 3:**
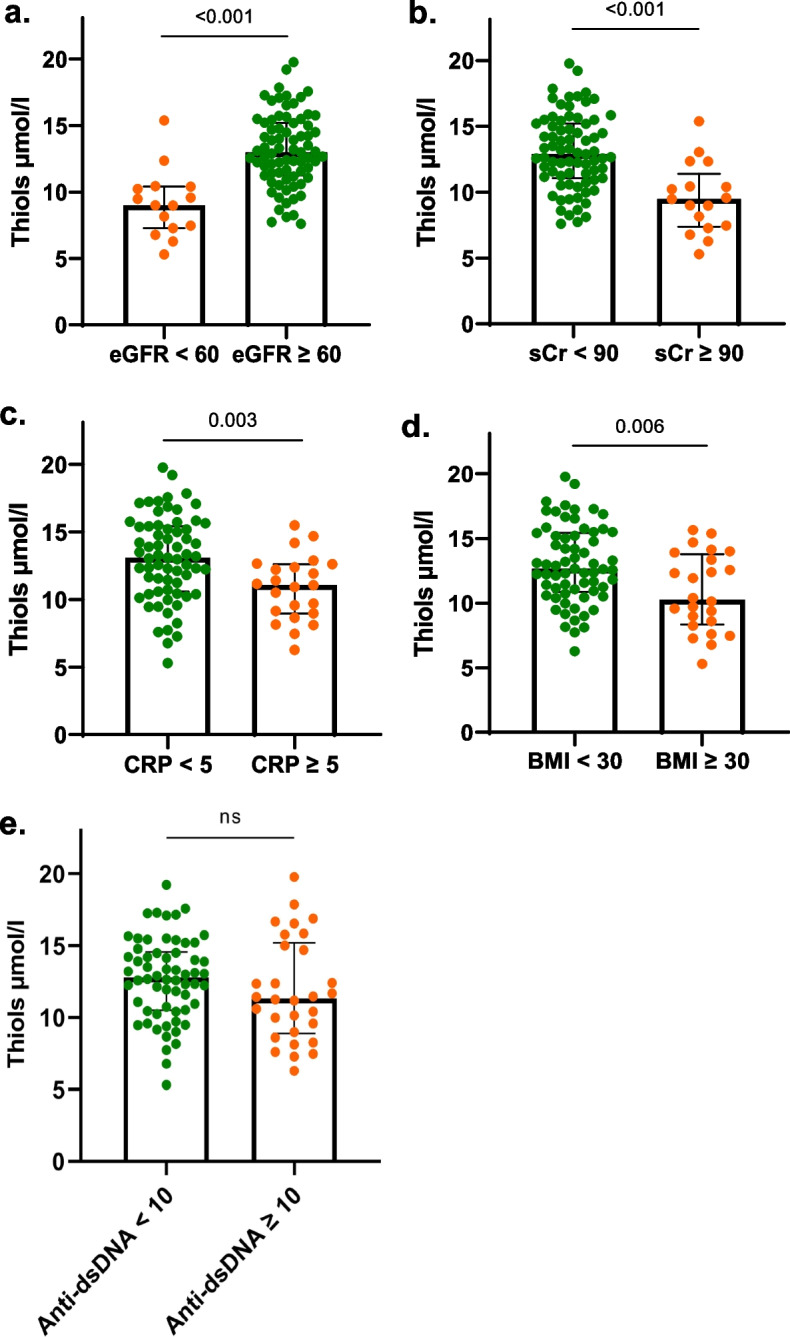
Comparison of free thiol levels in all SLE patients stratified for different clinical parameters. Legends 3. (**a**) Free thiol levels are compared between subgroups stratified for eGFR; (**b**) sCr; (**c**) CRP; (**d**) BMI; (**e**) anti-dsDNA. Line in each bar represents median with interquartile range; ns, not significant. eGFR, estimated glomerular filtration rate; sCr, serum creatinine; CRP, C-reactive protein; BMI, body mass index; Anti-dsDNA, anti-double stranded DNA

For all 100 SLE patients, NETs and free thiol levels were compared between subgroups stratified for clinically relevant cut-off values of clinical and biological parameters. Median levels of NETs were higher in SLE patients with CRP ≥ 5 mg/L compared to patients with CRP < 5 mg/L (*p* = 0.002). No significant differences were found between other subgroups, i.e. eGFR (cut-off 60 ml/min), complement 3 (C3, cut-off 0.9 g/L), complement 4 (C4, cut-off 0.1 g/L), anti-double stranded DNA (anti-dsDNA, cut-off 10 IU/ml) or neutrophils total amount (cut-off 7.10^9). Concerning the levels of free thiols, SLE patients with eGFR < 60 ml/min, sCr ≥ 90 µmol/L, CRP ≥ 5 mg/L, body mass index (BMI) ≥ 30 kg/m^2^ had lower median levels of free thiols, reflecting more OS (*p* < 0.001, < 0.001, 0.003, 0.006, respectively). No significant differences were found for patients with anti-dsDNA levels ≥ 10 IU/ml (Fig. 3).

### Logistic regression

Univariable logistic regression showed that age, disease duration, BMI, systolic blood pressure, proteinuria, number of neutrophils, CRP, eGFR, sCr, albumin (Alb), C3 levels and NETs were significantly associated with free thiols (Table 2). eGFR had the highest explained variance (R^2^ = 0.31). In multivariable logistic regression analysis for thiols, disease duration, BMI, systolic blood pressure, proteinuria, NETs, number of neutrophils, CRP, eGFR, Alb and C3 were included (Table 2). Of these variables, disease duration (OR -0.08, 95% CI -0.14 to-0.02), NETs (OR -0.05, 95% CI -0.09 to -0.02) and eGFR (OR 0.06, 95% CI 0.03 to 0.09) were identified as independent predictors for free thiols. The explained variance of this multivariable model was 44% (Table 2).

**Table 2:** Logistic regression with thiols as dependent variable with demographic, clinical and laboratory parameters

	R^2^	Univariable analysis		Multivariable analysis	
		B (95% CI)	*p-value*	B (95% CI)	*p-value*
Gender female (%)	0.019	1.173 (-0.612 to 2.958)	0.195		a
Age (year)	0.239	-0.105 (-0.145 to -0.066)	*0.000*		b
Disease duration (year)*	0.133	-0.106 (-0.165 to -0.047)	*0.001*	-0.082 (-0.144 to -0.020)	*0.010*
LN (yes/no)	0.038	-1.333 (-2.743 to 0.077)	0.064		a
SLEDAI (score)	0.011	0.229 (-0.231 to 0.689)	0.326		a
BMI (kg/m2)*	0.09	-0.169 (-0.285 to -0.052)	*0.005*		c
Prednisolone (yes/no)	0.041	-1.283 (-2.575 to 0.009)	0.052		a
Smoking (yes/no)**	0.002	0.302 (-1.178 to 1.781)	0.686		a
Alcohol (yes/no)**	0.004	0.423 (-1.181 to 2.028)	0.601		a
Systolic blood pressure (mmHg)	0.071	-0.053 (-0.093 to -0.013)	*0.010*		c
Diastolic blood pressure (mmHg)	0.007	-0.029 (-0.101 to 0.042)	0.414		a
NET (Au/ml)	0.067	-0.048 (-0.086 to -0.010)	*0.013*	-0.054 (-0.086 to -0.021)	*0.002*
Proteinuria (yes/no)*	0.059	-2.315 (-4.256 to -0.373)	*0.020*		c
Thrombocytes (10^9/L)*	0.004	0.003 (-0.006 to 0.012)	0.533		a
Hemoglobin (mmol/L)	0.042	0.726 (-0.001 to 1.452)	*0.050*		c
Lymphocytes total amount (10^9/L)	0.017	0.667 (-0.410 to 1.743)	0.222		a
Neutrophils total amount (10^9/L)	0.050	-0.285 (-0.546 to -0.024)	*0.033*		c
Leukocytes (10^9/L)	0.032	-0.212 (-0.457 to 0.033)	0.089		a
C-reactive protein (mg/L)*	0.067	-0.098 (-0.176 to -0.021)	*0.014*		c
eGFR (ml/min)	0.307	0.072 (0.049 to 0.095)	*0.000*	0.059 (0.033 to 0.085)	< *0.001*
Serum creatinine (µmol/L)	0.242	-0.054 (-0.074 to -0.034)	*0.000*		b
Albumin (g/L)**	0.057	0.194 (0.017 to 0.371)	*0.033*		c
Cholesterol (mmol/L)*	0.009	-0.325 (-1.028 to 0.377)	0.360		a
Triglyceride (mmol/L)¶	0.010	-0.384 (-1.529 to 0.761)	0.503		a
LDL-C (mmol/L)*	0.003	-0.230 (-1.072 to 0.612)	0.588		a
HDL-C (mmol/L)*	0.002	0.303 (-1.004 to 1.609)	0.647		a
ALAT (U/L)	0.005	0.016 (-0.029 to 0.062)	0.483		a
anti-dsDNA titer (IU/ml)	0.002	0.008 (-0.029 to 0.045)	0.672		a
Complement 3 (g/L)	0.107	-4.871 (-7.823 to -1.919)	*0.001*		c
Complement 4 (g/L)	0.032	-6.351 (-13.658 to 0.957)	0.088		a

*Abbreviations*: *LN* Lupus nephritis, *SLEDAI* the Systemic Lupus Erythematosus Disease Activity Index, *BMI* Body mass index, *NET* Neutrophil extracellular trap, *eGFR* estimated glomerular filtration rate, *LDL-C* Low-density lipoprotein-cholesterol, *HDL-C* High-density lipoprotein-cholesterol, *ALAT* Alanine aminotransferase, *anti-dsDNA* anti-double stranded DNA

a. The variable was not included in multivariable regression analysis due to a *p-value* of ≥ 0.050 in univariable regression analysis

b. Age and serum creatinine were not tested in multivariable regression analysis because disease duration and eGFR were included

c. Variables were not selected during multivariable regression analysis (*p* ≥ 0.050)

Missing data * ≤ 7%, **7–17%, ¶ > 17%; B, coefficient

Univariable logistic regression showed that CRP and high-density lipoprotein-cholesterol (HDL-C) were associated with NETs (R^2^ = 0.07, R^2^ = 0.05) (Supplementary Table 2). Multivariable logistic analysis for NETs was not performed, because of the low number of significant variables and relatively low explained variance in univariable regression analysis.

## Discussion

In this cross-sectional analysis of SLE patients, levels of NETs, measured as MPO-DNA complexes were increased in non-LN and LN patients compared to HCs. Levels of free thiols were not significantly different between the groups. However, NETs and free thiols were inversely correlated in SLE patients suggesting that lower levels of free thiols reflecting enhanced OS is related to higher levels of NETs. Furthermore, we found that free thiols were independently associated with disease duration, NETs levels and eGFR in all SLE patients.

Although there are more reports on the role of NETs or OS in SLE, the present study is the first one to investigate the relationship between NETs and OS in SLE. A previous study from our group showed that NETs were increased in SLE and also in incomplete SLE patients, while levels of low-density granulocytes, which are regarded as important producers of NETs were increased in both SLE and iSLE [10]. Even when the disease is in remission, NETs levels remain elevated, which seems to be a result of multifactorial mechanisms, suggesting that neutrophil dysfunction, induction of NETs formation, and impaired clearance of NETs consistently occur in SLE [14, 29]. We did not find a difference in NETs levels between SLE patients with or without a history of LN, which may be due to long disease duration and remission status of the patients. Recently, several studies have addressed the impact of NETs on SLE clinical outcomes. A prospective study showed that reduced degradation of NETs was observed in SLE patients and was linked to disease activity. Decreased NET degradation was associated with glomerulonephritis, low complement levels, more severe clinical manifestations and elevated antibodies against histones and DNA [30]. Another study showed that elevated elastase-DNA and high-mobility group box 1 (HMGB1)-DNA complexes were associated with worse renal outcomes in active LN, especially in patients with proliferative LN. In addition, patients with higher levels of NETs at baseline were more likely to experience flares and severe renal impairment over 24 months following LN, implying that NETs may serve as predictors of high risk for disease activity [31].

In our study, NETs were correlated with CRP in SLE patients. In general, CRP is an acute-phase protein that increases a lot in response to inflammation. CRP also slightly increases in coronary heart disease, so, it is not a specific marker involved in SLE. However, a relation between CRP and NETs was also described by others [32] indicating that chronic low grade inflammation, of which it is known that it increase OS, might increase the levels of NETs via OS.

Although the pathogenesis of LN is complex, OS has been found to play an important role in the development of renal impairment, with ROS/RNS-mediated NETs generation observed to cause renal epithelial cell damage [33]. Our previous study has shown that free thiol levels were lower, reflecting elevated OS in SLE patients with active nephritis, and were correlated with disease activity over time [19]. The present study demonstrates that SLE patients with or without a history of nephritis in remission with long disease durations are likely to be characterized by OS attenuation, as levels of free thiols are comparable to those of healthy individuals. We did demonstrate that free thiols were independently associated with eGFR which suggests that increased OS is linked to a higher risk of decrease renal function. This is consistent with a previous finding that extracellular free thiols were positively associated with eGFR, and inversely associated with BMI and CRP levels in chronic kidney disease (CKD) patients [21]. Lower levels of thiols indicate an unfavorable redox state and are associated with risk factors such as ageing, disease duration, obesity and hypertension [22]. A possible explanation for the relation of free thiols with eGFR could be that extracellular free thiol groups mainly consist of cysteine-based proteins, of which albumin is the most abundant protein. In addition, free thiols are present within low-molecular-weight proteins, such as cystatin C and glutathione [34, 35], therefore, renal function appears to be connected with free thiols.

It has been demonstrated that ROS/ RNS is required for NETs formation, because it increases neutrophil membrane permeability, driving chromatin decondensation and promoting morphological changes during NETosis. An interesting review summarized that neutrophil-derived ROS and proteases contribute to tissue damage, modification of proteins, lipids and DNA, along with the dysregulation of redox homeostasis during autoimmune responses [8]. In line with our findings, another study showed that OS measured by dihydrorhodamine, which measures the oxidative burst of neutrophils, was associated with release of NET-associated elastase in pediatric SLE. In accordance with their findings, however, we did not observe a correlation with anti-dsDNA or C3 levels [36]. These findings need to be further assessed as NETs were experimentally measured in plasma and in situ studies of NETs in human kidneys are still lacking. Interestingly, NETs levels were found to be negatively associated with HDL-C in our study. In accordance, a previous lupus-prone mice study reported that pharmacologic inhibition of NETs in vivo potentially decreased levels of proinflammatory oxidized HDL [37]. Therefore, the presence of increased OS as well as increased NETosis might enhance HDL oxidation promoting atherosclerosis.

Inhibiting NETosis might be beneficial for preventing the effects of NETs in inflammation, such as occurrence of autoantigens and autoreactivity in SLE [38]. Several potential targets for either inhibiting NETs formation or accelerating clearance of NETs have been described. A preclinical study demonstrated that therapeutic anti-citrullinated protein antibody (tACPA) prevented disease symptoms through NETs inhibition or potentially via activating uptake and digestion of pre-NETs and NETs by macrophages in both murine models of neutrophil-mediated inflammatory diseases and in vitro assays [39].

Inhibiting OS might also result in less NETs formation in diseases. Nuclear factor-erythroid 2-related factor 2 (Nrf2) is a transcription factor that plays a central role in inducing antioxidant responses. In antineutrophil cytoplasmic antibody (ANCA)-associated vasculitis (AAV), pharmacological activation of Nrf2 has been reported to protect endothelial cells and ameliorate glomerulonephritis by inhibiting ROS-induced NETs formation in an AAV mice model [40]. Another study also reported that dietary taxifolin exerted potent effects by suppressing neutrophil hyperactivity and normalizing inflammatory responses, partially through activation of Nrf2 signalling pathways in neutrophils in vivo in a thrombo-inflammatory mouse model of lupus [41]. Another study demonstrated that human serum albumin-fused thioredoxin (HSA-Trx), a protein thiol-disulfide oxidoreductase with a long half-life, suppressed NETs formation via reducing ROS production in aerosol-induced lung injury in mice [42].

The strength of this study is that we analyzed correlations between a biomarker for OS, namely thiols, and NETs levels under clinical conditions in lupus. Limitations of the study include the relatively small number of patients and the fact that all patients were in remission. Therefore, not enough power was present to explore the relationship between NETs formation and OS with other possible confounding factors such as cardiovascular/atherosclerotic risk factors, diet, disease activity or different immunosuppressive therapies. Our findings also encourage further studies unravelling the potential link between NETs and OS and cardiovascular morbidity in SLE.

## Conclusion

In conclusion, NETs formation was increased in SLE patients with and without renal involvement, whereas free thiol levels were not statistically different in quiescent SLE compared to HCs. Levels of NETs and free thiols were significantly negatively correlated in SLE patients, supporting a possible role of OS in the formation of NETs in SLE. Therefore, decreasing NETs levels, possibly via reducing OS, might be an adjuvant therapy for treatment of SLE. However, large-scale follow-up studies are required to obtain more defined conclusions.

## Supplementary Information

Supplementary Material 1.

## Data Availability

No datasets were generated or analysed during the current study.

## Abbreviations

SLE: Systemic lupus erythematosus
LN: Lupus nephritis
CKD: Chronic kidney disease
HCs: Healthy controls
NETs: Neutrophil extracellular traps
SLEDAI: Systemic Lupus Erythematosus Disease Activity Index
eGFR: Estimated glomerular filtration rate
sCr: Serum creatinine
CRP: C reactive protein
BMI: Body mass index
TG: Triglyceride
HDL-C: Hgh-density lipoprotein-cholesterol
Alb: Albumin
Hb: Haemoglobin
anti-dsDNA: Anti-double stranded DNA
OS: Oxidative stress
ROS: Reactive oxygen species
RNS: Reactive nitrogen species
HSA-Trx: Human serum albumin-fused thioredoxin
HMGB1: High-mobility group box 1
NSAID: Non-steroidal anti-inflammatory drugs
tACPA: Therapeutic anti-citrullinated protein antibody
Nrf2: Nuclear factor-erythroid 2-related factor 2
AAV: Antineutrophil cytoplasmic antibody-associated vasculitis
