# Supplementary material for: Neutrophil extracellular traps and oxidative stress in systemic lupus erythematosus patients with and without renal involvement
Source: Arthritis Res Ther. 2024 Dec 19;26:220. doi: 10.1186/s13075-024-03454-y (PMC11658285; doi:10.1186/s13075-024-03454-y)
Supplement: Supplementary file 1 — Supplementary Material 1. [file 13075_2024_3454_MOESM1_ESM.pdf]

Supplementary

Supplementary 1. The distribution of different classes of lupus nephritis (LN)

Classification of LN	Number of patients
I	1
II	1
III	5
IV	14
V	2
III/IV	1
III/V	2
IV/V	1

Supplementary 2. Logistic regression with Log10(NETs+1) as dependent variable with demographic, clinical and laboratory parameters

			Univariable analysis
	R2	B (95% CI)	p-value
Gender female (%)	0.009	0.116(-0.135 to 0.366)	0.363
Age (year)	0.000	0.000 (-0.006 to 0.007)	0.877
Disease duration (year)*	0.001	-0.002 (-0.010 to 0.007)	0.723
LN (yes/no)	0.000	0.011 (-0.190 to 0.211)	0.917
SLEDAI (score)	0.006	0.024 (-0.040 to 0.088)	0.461
BMI (kg/m2)**	0.002	0.003 (-0.014 to 0.020)	0.702
Prednisolone (yes/no)	0.003	0.045 (-0.139 to 0.229)	0.629
Smoking (yes/no)**	0.033	0.173 (-0.038 to 0.384)	0.107
Alcohol (yes/no)**	0.003	0.058 (-0.168 to 0.284)	0.612
Systolic blood pressure (mmHg)	0.003	-0.002 (-0.007 to 0.004)	0.593
Diastolic blood pressure (mmHg)	0.008	-0.004 (-0.014 to 0.006)	0.381
Proteinuria (yes/no)*	0.033	0.242 (-0.034 to 0.518)	0.085
Thrombocytes (10^9/L)*	0.005	0.000 (-0.002 to 0.001)	0.523
Hemoglobin (mmol/L)	0.000	-0.010 (-0.113 to 0.094)	0.850
Lymphocytes total amount (10^9/L)	0.000	0.01 (-0.142 to 0.162)	0.901
Neutrophils total amount (10^9/L)	0.029	0.031 (-0.006 to 0.068)	0.101
Leukocytes (10^9/L)	0.030	0.029 (-0.005 to 0.063)	0.095
C-reactive protein (mg/L)*	0.072	0.014 (0.004 to 0.025)	0.010
eGFR (ml/min)	0.000	‘0.000 (-0.004 to 0.004)	0.885
Serum creatinine (µmol/L)	0.001	0.000 (-0.004 to 0.003)	0.772
Albumin (g/L)**	0.000	0.001 (-0.025 to 0.026)	0.955

Cholesterol (mmol/L)*	0.005	0.034 (-0.064 to 0.133)	0.491
Triglyceride (mmol/L)¶	0.007	-0.047 (-0.221 to 0.126)	0.587
LDL-C (mmol/L)*	0.024	0.088 (-0.029 to 0.204)	0.138
HDL-C (mmol/L)*	0.049	-0.194 (-0.372 to -0.016)	0.033
ALAT (U/L)	0.003	-0.002 (-0.008 to 0.005)	0.621
anti-dsDNA titer (IU/ml)	0.002	0.001 (-0.004 to 0.006)	0.635
Complement 3 (g/L)	0.023	0.317 (-0.115 to 0.750)	0.148
Complement 4 (g/L)	0.005	0.367 (-0.669 to 1.404)	0.483

Abbreviations and missing data, see Table 2.
